# A fused multi-subfrequency bands and CBAM SSVEP-BCI classification method based on convolutional neural network

**DOI:** 10.1038/s41598-024-59348-1

**Published:** 2024-04-14

**Authors:** Dongyang Lei, Chaoyi Dong, Hongfei Guo, Pengfei Ma, Huanzi Liu, Naqin Bao, Hongzhuo Kang, Xiaoyan Chen, Yi Wu

**Affiliations:** 1 College of Electric Power, Inner Mongolia University of Technology, Hohhot, 010080 China; 2 Intelligent Energy Technology and Equipment Engineering Research Centre of Colleges and Universities in Inner Mongolia Autonomous Region, Hohhot, 010051 China; 3 Engineering Research Center of Large Energy Storage Technology, Ministry of Education, Hohhot, 010080 China; 4 Inner Mongolia Academy of Science and Technology, Hohhot, 010010 China

**Keywords:** Computational biology and bioinformatics, Electroencephalography - EEG

## Abstract

For the brain-computer interface (BCI) system based on steady-state visual evoked potential (SSVEP), it is difficult to obtain satisfactory classification performance for short-time window SSVEP signals by traditional methods. In this paper, a fused multi-subfrequency bands and convolutional block attention module (CBAM) classification method based on convolutional neural network (CBAM-CNN) is proposed for discerning SSVEP-BCI tasks. This method extracts multi-subfrequency bands SSVEP signals as the initial input of the network model, and then carries out feature fusion on all feature inputs. In addition, CBAM is embedded in both parts of the initial input and feature fusion for adaptive feature refinement. To verify the effectiveness of the proposed method, this study uses the datasets of Inner Mongolia University of Technology (IMUT) and Tsinghua University (THU) to evaluate the performance of the proposed method. The experimental results show that the highest accuracy of CBAM-CNN reaches 0.9813 percentage point (pp). Within 0.1–2 s time window, the accuracy of CBAM-CNN is 0.0201–0.5388 (pp) higher than that of CNN, CCA-CWT-SVM, CCA-SVM, CCA-GNB, FBCCA, and CCA. Especially in the short-time window range of 0.1–1 s, the performance advantage of CBAM-CNN is more significant. The maximum information transmission rate (ITR) of CBAM-CNN is 503.87 bit/min, which is 227.53 bit/min-503.41 bit/min higher than the above six EEG decoding methods. The study further results show that CBAM-CNN has potential application value in SSVEP decoding.

## Introduction

Brain computer interface (BCI) is a new form of human–computer interaction that connects the human brain to external devices^1,2^. BCI technology has been widely used in rehabilitation engineering^3^, fatigue detection^4^, and smart home^5^. With the development of BCI technology, many typical paradigms have emerged, such as steady-state visually evoked potential (SSVEP)^6^, P300^7^, and motor imagery (MI)^8^. When the subject is stimulated by a specific frequency of vision, the visual cortex of the brain produces a continuous electrical response signal related to the stimulus frequency, which is called SSVEP^9^. In the SSVEP-BCI system, each specific stimulus frequency can be mapped to a specified control instruction, and the SSVEP signals are reversely decoded by a method designed to obtain the classification result of the control command^10^. SSVEP has attracted the attention of many scholars and been widely used because of its advantages of high information transmission rate (ITR), high signal-to-noise ratio (SNR), and less training requirement^11–15^.

Traditional target recognition methods for SSVEP paradigm include the continue wavelet transform (CWT)^16^ and canonical correlation analysis (CCA)^17^. CWT method extracts features of the SSVEP signals in both time and frequency domains. Moreover, the method relies on prior knowledge to extract several frequency bands of interest, and then uses wavelet coefficients as features for classification. The core of CWT is to choose the appropriate mother wavelet, and different mother wavelets usually produce different classification results. The CCA is widely used in SSVEP-BCI systems due to its advantages of fast computational speed and robustness. The basic idea of CCA is to quantitatively calculate the correlation between the reference signal constructed by sine and cosine and the EEG signals to be detected, and then the frequency of the stimulus target is identified using the maximum correlation coefficient. Although CCA and CWT target recognition methods have different characteristics and both of them can achieve certain effectiveness, the accuracies of the two methods are still in a relatively low level. To improve the accuracy of SSVEP task classification, researchers have proposed many improved methods of CCA. For example, a method of combining multivariate variational mode decomposition (MVMD) with CCA was proposed to improve the detection and classification ability of SSVEP signals. In 2017, Nakanishi et al.^18^ proposed task-related component analysis (TRCA), which can maximize the reproducibility among multiple trials of SSVEP signals and improve their SNR. Therefore, the method is especially suitable for the classification task of time-locked signals such as SSVEP. Chen et al. proposed a filter bank canonical correlation analysis (FBCCA) method, which combined the fundamental and harmonic frequency components to apply CCA to the filter multi-subfrequency bands of EEG signals. The FBCCA method can improve the ITR and accuracy of SSVEP-BCI. With the development of machine learning theory, more and more machine learning models have been applied to the target classification task of SSVEP-BCI, including linear discriminant analysis (LDA)^19^, Gaussian naive Bayes (GNB)^20^, recursive Bayes (RB)^21^, and supporting vector machine (SVM)^22^. The above traditional methods have significant advantages in solving different specific classification problems. However, the features extracted and processed by the above methods are single, and the coding ability for advanced features is insufficient. Especially when dealing with the classification of complex EEG signals, the accuracy and ITR need to be improved.

In recent ten years, deep learning methods have shown great capabilities in image processing, speech recognition, and natural language processing^23–25^. Because of the unique ability of deep learning in dealing with nonlinear, non-stationary, and random signal modeling, deep learning networks, such as convolution neural network (CNN), have been gradually applied to the field of EEG modeling and classification, and achieved remarkable results^26,27^. The CNN method learns features with its own model structure and does not require manual feature design. Moreover, CNN has better adaptive and self-learning capabilities when processing EEG signals, and has better generalisation capabilities than traditional methods. In 2017, Kwak et al.^28^ proposed a CNN-based SSVEP classifier in dynamic environment, and the accuracy of SSVEP signals classification reached 94.03%. The CNN method can achieve better performance than traditional machine learning methods in signal feature characterization and learning. However, the role of CNN in the characterization and enhancement of key features still has a space of improvement and needs to be further strengthened. In deep learning networks, the introduction of attention mechanisms can match corresponding weights based on the importance of different features in the network. The attention mechanism can enhance the contribution of some important key features while weakening the contribution of secondary features. Thus, the mechanism can serve to further feature extraction and enhance the performance of the model^29,30^. At present, many types of attention mechanism models have been proposed. For example, squeeze-and-excitation network (SENet) adaptively adjusts the influence between channels by feature recalibration method to make more effective use of features^31^. The efficient channel attention network (ECANet) avoids the influence of SENet dimension reduction through one-dimensional convolution cross-channel interaction^32^. Spatial transformer networks (STN) gain better robustness by training the spatial transformation corresponding to a specific input^33^. The above attention models only strengthen the features unilaterally from the respects of space or channel, therefore the features represented are partial. The convolutional block attention module (CBAM) takes into account the characteristics of both space and channel, and infers the attention map in turn through the two independent dimensions of channel and space. Then the attention map is multiplied by the input feature map for further adaptive feature optimization, which can effectively improve the performance of the deep learning model.

In this paper, a fused multi-subfrequency bands and CBAM classification method based on CNN (CBAM-CNN) is proposed. The multi-subfrequency bands can extract the feature information of SSVEP signals more comprehensively. Moreover, the embedded CBAM uses both spatial and channel attention to improve the feature representation ability of deep learning networks^34^. The CBAM-CNN model structure proposed in this paper has higher accuracy and ITR of SSVEP signals compared with other classical methods under short-time windows. Especially, the CBAM-CNN model structure has better self-adaptive capability.

## Proposed CBAM-CNN method

**Figure 1:**
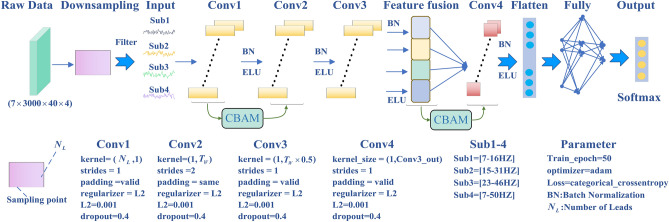
The network architecture of CBAM-CNN.

The proposed CBAM-CNN model provides a new method for identifying SSVEP-BCI tasks. The model incorporates more abundant feature information of SSVEP signals. At the same time, the embedded CBAM uses space and channel attention to further improve the feature representation ability of deep learning networks. As shown in Fig. 1, the CBAM-CNN structure consists of a down sample layer, an input layer, a convolution layer, a feature fusion layer, a CBAM layer, a flatten layer, a fully connected layer, and an output layer. The raw data is $7 \times 3000 \times 40 \times 4$, where 7 is the number of leads, 3000 is the number of sampling points in one experiment per stimulation frequency, and 40 is the number of experiments per stimulation frequency. 4 is the number of stimulation frequencies. The CBAM-CNN network needs to process the data in the form of $7 \times 24000$ before entering the layer.

The first layer of CBAM-CNN network structure is to reduce the sampling frequency of the original EEG data. Downsampling is used to adjust the sampling frequency of the original data from 1000 to 500 Hz. The input layer acquires multi-subfrequency bands signals by means of Butterworth filters. The low SNR of subfrequency band signals leads to a reduction in the effectiveness of signal analysis and feature extraction. The SSVEP signal in the frequency band above 50 Hz has a low SNR. Therefore, the CBAM-CNN method does not use multi-subfrequency bands information above 50 Hz. The frequency range of multi-subfrequency bands are 7–16 Hz, 15–31 Hz, 23–46 Hz and 7–50 Hz, respectively. Among them, the subfrequency bands 7–16 Hz, 15–31 Hz and 23–46 Hz are selected according to the first harmonic, second harmonic and third harmonic of the stimulation frequency. Each harmonic has a complete feature information. The subfrequency bands 7–50 Hz represent comprehensive feature information of available bands. The multi-subfrequency bands signals are set up to extract the SSVEP signals characteristics more fully in temporal information and spatial information. The multi-subfrequency bands signals of four subfrequency bands are used as the initial input of the convolution layer. Then the signals are transformed to four refined features via the sequent layers of Conv1, Conv2, Conv3, and CBAM. After that, the feature fusion layer fuses the four refined features of its upper layer. In addition, the CBAM-CNN method embeds a second CBAM module between the feature fusion layer and Conv4 to enhance the attention to important features with focus in the spatial and channel dimensions.

Conv1, Conv2, Conv3, and Conv4 are four convolution layers of CBAM-CNN network. The convolution kernel of Conv1 is $N_{L} \times 1$. The $N_{L}$ represents the number of leads. Conv1 outputs the temporal information of SSVEP signals. The convolution kernel of the second convolution is $1 \times T_{W}$, where $T_{W}$ is sampling period after downsampling. Conv2 outputs the spatial information of SSVEP signals. Each convolutional layer is followed by a batch normalization (BN) layer to normalize the SSVEP data. The BN layer can convert the current input data into a standard normal distribution with a mean of 0 and a variance of 1, thus accelerating the speed of model convergence, controlling gradient explosion, preventing gradient disappearance and overfitting. CBAM-CNN adopts an ELU function, an unsaturated activation function, as its activation function, and the strength of ELU activation function is its ability to alleviate gradient disappearance and its robustness to noise. The feature fusion layer fuses four frequency band signals, and then important features can be extracted via a second CBAM and Conv4 sequentially. Obviously, the output of the Conv4 layer is high-dimensional and cannot be transmitted to a final fully connected layer. So, the high-dimensional data is converted into one-dimensional data by flatten layer to be used as the input to the fully connected layer.

**Figure 2:**
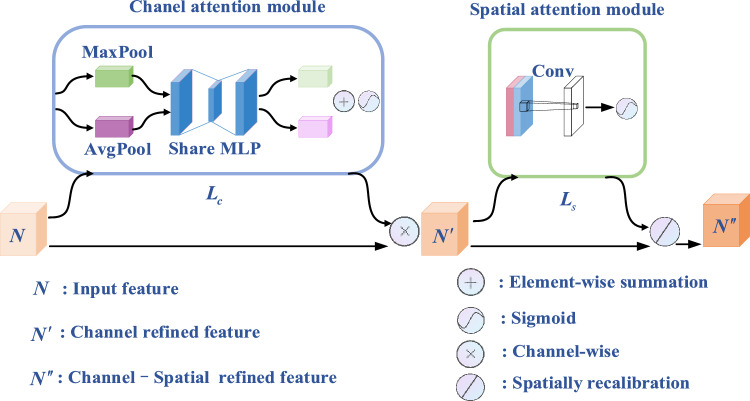
The network architecture of CBAM.

Lightweight module CBAM is a kind of attention mechanism of feedforward convolutional neural network. The important functions of CBAM are filtering irrelevant information, solving the problem of information overload, and improving the accuracy of task processing. CBAM completes the channel attention processing on the input feature map, and then carries out the spatial attention mechanism. Thus, these operations can strengthen the region of interest from both channel and spatial dimensions and obtain an inferred attention map. Then, the attention map is multiplied by the input feature map for adaptive feature refinement. The network structure of CBAM is shown in Fig. 2.

Channel attention module mainly includes global average pooling module, global maximum pooling module, and shared MLP module. The output of shared MLP is fused by element-wise summation, and then ${\varvec{L}}_{{\varvec{c}}} \in {\varvec{R}}^{C \times 1 \times 1}$ is obtained by a sigmoid activation. ${\varvec{L}}_{{\varvec{c}}}$ can be expressed as

1 $${\varvec{L}}_{{\varvec{c}}} \left( {\varvec{N}} \right) = \sigma \left( {MLP\left( {AvgPool\left( {\varvec{N}} \right)} \right) + MLP\left( {MaxPool\left( {\varvec{N}} \right)} \right)} \right),$$

where $\sigma$ denotes the sigmoid function, and ${\varvec{N}} \in {\varvec{R}}^{C \times H \times W}$ denotes the input feature map and ${\varvec{L}}_{{\varvec{c}}}$ represents 1D channel attention map.

Channel refined feature ${\varvec{N}}^{\prime }$ is obtained using ${\varvec{L}}_{{\varvec{c}}}$ and ${\varvec{N}}$ by channel-wise as (2).

2 $${\varvec{N}}^{\prime } = {\varvec{L}}_{{\varvec{c}}} \left( {\varvec{N}} \right) \otimes {\varvec{N}}.$$

where $\otimes$ represents the element-wise multiplication between the feature weight of each channel and ${\varvec{N}}$.

${\varvec{L}}_{{\varvec{s}}} \in {\varvec{R}}^{1 \times H \times W}$ is obtained by

3 $${\varvec{L}}_{{\varvec{s}}} \left( {{\varvec{N}}^{\prime } } \right) = \sigma \left( {f^{{Z^{\prime}}} \left( {\left[ {AvgPool\left( {{\varvec{N}}^{\prime } } \right);MaxPool\left( {{\varvec{N}}^{\prime } } \right)} \right]} \right)} \right),$$

where $Z^{\prime }$ represents the convolution kernel size, and ${\varvec{L}}_{{\varvec{s}}}$ denotes 2D spatial attention map. In (1), the dimensions of the AvgPool and MaxPool outputs are both 1D. In (3), the dimensions of the AvgPool and MaxPool outputs are both 2D.

${\varvec{N}}^{\prime }$_gets the final redefined feature_ ${\varvec{N}}^{\prime \prime }$_through spatial attention module._ ${\varvec{N}}^{\prime \prime }$_can be expressed as_

4 $$\user2{N^{\prime\prime}} = {\varvec{L}}_{{\varvec{s}}} \left( {\user2{N^{\prime}}} \right)\emptyset \user2{N^{\prime}}.$$

where $\emptyset$ represents the element-wise multiplication between the spatial feature weights and $\user2{N^{\prime}}$.

The parameters of CBAM-CNN are optimized by Adam algorithm. Traditional random gradient descent maintains a single learning rate, however Adam algorithm updates the weights of neural network based on training data iteration, with adjustable learning rate and strong adaptability. Additionally, cross entropy function is selected as the loss function of CBAM-CNN.

## Experimental dataset

**Figure 3:**
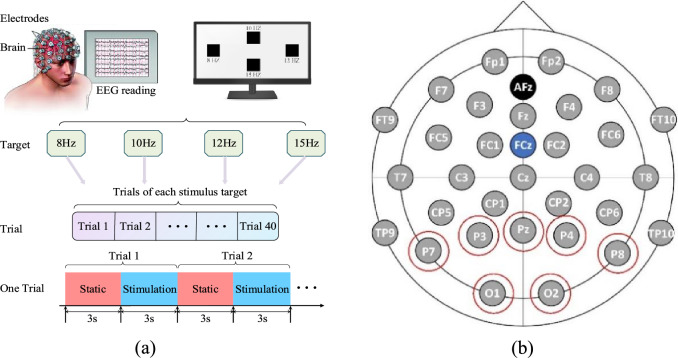
Experimental setup. **a** Experimental timing diagram of IMUT EEG data recording. **b** EEG cap with the 32 lead positions for the equipment from BP Inc., Germany^35^.

EEG dataset from Inner Mongolia University of Technology (IMUT) is recoded by a 32-lead EEG acquisition device from Brain Products (BP) Inc., Germany. The visual stimulus interface is realized by Matlab-Psychtoolbox toolbox. The stimulus frequencies were set to 8 Hz, 10 Hz, 12 Hz, and 15 Hz respectively. The data acquisition process is shown in Fig. 3a, the trial first displays the static interface for 3 s, and then stimulates the color block on the screen to blink for 3 s, where the static interface is used for subjects to rest. The subject needs to complete 160 trials with four visual stimulus frequencies according to the above process. In this study, both IMUT and Tsinghua University (THU) EEG datasets are used to verify the effectiveness of the method. IMUT and THU datasets contain EEG data of 25 subjects and 35 subjects respectively, and each subject has normal vision after correction.

In Fig. 3b, lead O represents the occipital area of the brain and lead P indicates the parietal lobe area of the brain. The occipital area of the brain is the visual cortex center, which is responsible for the visual processing function. Therefore, the target lead of SSVEP signals is usually selected in the occipital area of the brain. In addition, the parietal lobe area is mainly responsible for the integration of spatial information, visual information, and somatosensory information processing. The occipital and parietal area is the brain functional area related to SSVEP signals. The occipital and parietal area contains only 7 leads (O1, O2, P3, P4, PZ, P7, P8). In this study, the multi-lead SSVEP signals contain seven leads from occipital and parietal area (O1, O2, P3, P4, PZ, P7, P8).

Actually, THU dataset is public and authoritative. It contains 35 subjects, including 17 females and 18 males. The frequency range of data acquisition experiment is 8–15.8 Hz and the frequency interval is 0.2 Hz, with a total of 40 frequencies. In order to facilitate visual gaze and avoid visual fatigue during stimulation, there is a one-minute rest time between two consecutive visual stimuli. In the SSVEP signal acquisition experiment, the signal acquisition processes of THU and IMUT datasets are basically the same. In the study, the selected electrodes and stimulation frequency are also the same.

## Performance evaluation

The evaluation metrics of the aforementioned methods used in this study included accuracy, ITR, recall, precision, and macro-F1.

The accuracy describes the proportion of correct prediction to the total sample, which can be expressed as

5 $$Accuracy = \frac{TP + TN}{{TP + FP + FN + TN}},$$

where $TP$ indicates the number of samples that are actually positive and the predicted result is also positive. $TN$ denotes the number of samples that are actually negative and the predicted result is also negative. $FP$ represents the number of samples that are actually negative and the predicted result is positive. $FN$ is the number of samples that are actually positive and the predicted result is negative. The current actual stimulation frequency is a positive sample. Not the current actual stimulation frequency is a negative sample.

The ITR represents the amount of information output by the system per unit time, which can be obtained by (6). The higher the ITR is, the better the real-time performance of SSVEP-BCI system is.

6 $$ITR = \frac{60}{{\tilde{T}}} \times (log_{2} r + mlog_{2} m + \left( {1 - m} \right)log_{2} \left[ {\frac{{\left( {1 - m} \right)}}{(r - 1)}} \right]),$$

where $m$ is accuracy, $r$ is the number of classified categories, and $\tilde{T}$ is the ingle target selection time.

The recall indicates the proportion of the correctly predicted samples in the actually positive samples. The metric is used to evaluate the detection coverage of the detector for all targets to be detected, and the expression is

7 $$Recall = \frac{TP}{{TP + FN}}.$$

The precision shows the proportion of correctly predicted positive samples to all predicted positive samples, which can be expressed as

8 $$Precision = \frac{TP}{{TP + FP}}.$$

The F1-score is the harmonic average of precision and recall. The F1-score can be more accurate and balanced to evaluate the performance of the model, which is a common dichotomous evaluation metric. Because the experiment in this paper sets four classification targets, the multi-classification metric macro-F1 is used to evaluate the model performance, which can be calculated by

9 $$Macro{ - }F1 = \frac{{\sum\nolimits_{i = 1}^{\kappa } {F1{ - }score_{i} } }}{\kappa },$$

where $F1{ - }score_{i}$ is the $F1{ - }score$ of the $i$-th classification, and $\kappa$ is the number of classification targets. $F1{ - }score_{i}$ can be calculated by

10 $$F1{ - }score_{i} = 2 \times \frac{{Recall_{i} \times Precision_{i} }}{{Recall_{i} + Precision_{i} }},$$

where $Recall_{i}$ is the $Recall$ of the $i$-th classification, and $Precision_{i}$ is the $Precision$ of the $i$-th classification.

## Experimental results

To verify the effectiveness of the algorithm, the proposed CBAM-CNN method is compared with six existing methods. The comparison methods are as follows: canonical correlation analysis (CCA)^36,37^, FBCCA, CCA-Gaussian naive Bayes (GNB)^38^, CCA-Supporting Vector Machine (SVM)^39^, CCA-Continue Wavelet Transform (CWT)-SVM^40,41^, and CNN. According to the comparison of experimental results of different kernels of SVM, SVM kernel is set as linear kernel for an optimal performance. CBAM-CNN, SVM-based and other comparison algorithms all have a division ratio of 9:1 between the training set and the test set.

Among them, CCA-SVM uses CCA to extract signal features and employ SVM as classifier. The feature extraction of CCA-CWT-SVM is completed jointly by CCA and CWT, and the target classification is finally completed by SVM At the same time, IMUT and THU EEG datasets are used to evaluate the performance of these methods. Due to the high real-time performance of SSVEP-BCI system, the recognition accuracy of related research below 2 s is low at present. Therefore, we choose 0.1, 0.5, 1, 1.5 and 2 s evenly within 2 s to study. SSVEP EEG signal decoding algorithm has good performance at 1.5 and 2 s.

**Figure 4:**
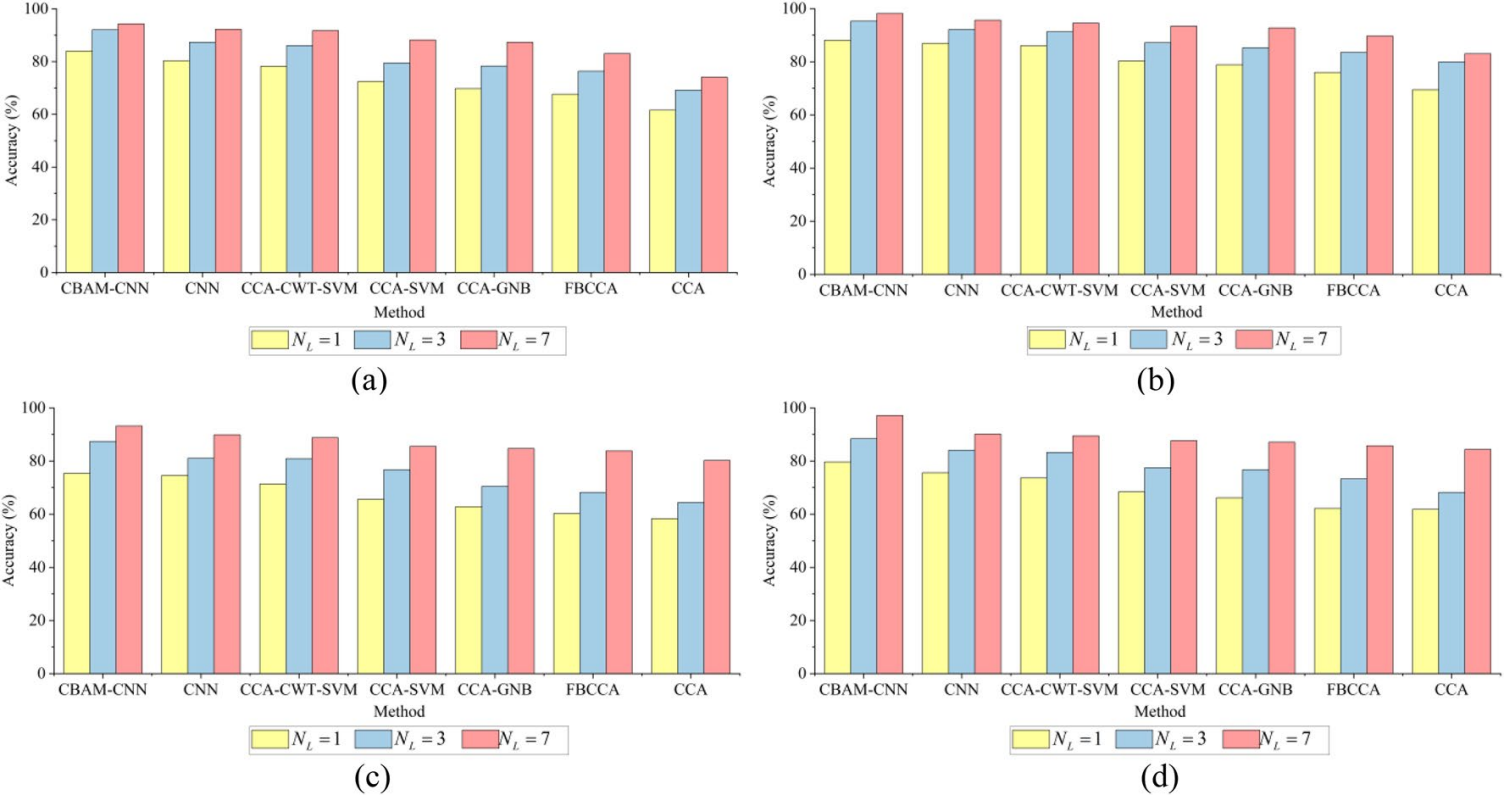
The accuracies of different methods under different $N_{L}$. **a** 1.5 s time window, IMUT dataset. **b** 2 s time window, IMUT dataset. **c** 1.5 s time window, THU dataset. **d** 2 s time window, THU dataset.

Because the feedback of SSVEP signals occurs in the occipital area of the brain, the signals of two leads within the occipital area and five surrounding leads were selected as the set of leads to be analyzed in this paper. The subsets $N_{L} = 1$ (O2), $N_{L} = 3$ (O1, O2, P3), $N_{L} = 7$ (O1, O2, P3, P4, PZ, P7, P8) are selected as different lead combinations to study the influence of $N_{L}$ on SSVEP signals recognition. The time window of the collected SSVEP signals is set to 1.5 s and 2 s, respectively. Figure 4 shows the test results of different methods, such as CCA, FBCCA, CCA-SVM, CCA-DWT-SVM, CCA-GNB, CNN, and CBAM-CNN, on IMUT dataset and THU dataset. Figure 4a and b show the test results of IMUT dataset, Fig. 4c and d show the test results of THU dataset. The results show that the accuracies of the seven methods increase with the increase of $N_{L}$ in the selected occipital region. When the maximum $N_{L}$ are 7, the accuracy of each method reaches the maximum. At the same time, the CBAM-CNN method is significantly superior to the other six comparison methods under different time windows and lead sets.

**Figure 5:**
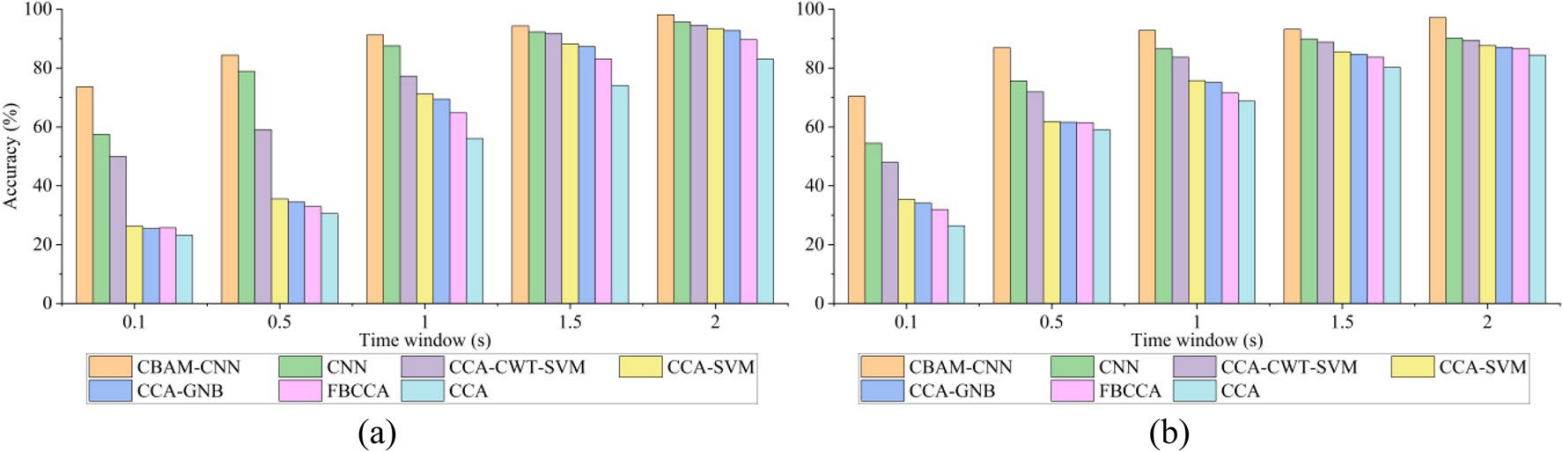
The accuracies of different methods under different time windows. **a** IMUT dataset. **b** THU dataset.

As shown in Fig. 5a, the accuracies of the different methods increase with the increase of time window in IMUT dataset. The accuracy of CBAM-CNN is significantly better than those of the other six methods under any time window. The accuracy of CBAM-CNN reaches the maximum of 98.13% under the 2 s time window. Within 0.1–1 s time window, the accuracy of CBAM-CNN is 3.63–16.17% higher than that of CNN, 13.97–25.38% higher than that of CCA-CWT-SVM, 20.04–48.85% higher than that of CCA-SVM, 21.80–49.94% higher than that of CCA-GNB, 26.42–47.89% higher than that of FBCCA, and 35.17–53.88% higher than that of CCA. Within 1–2 s time window, the accuracy of CBAM-CNN is 2.01–3.63% higher than that of CNN, 2.54–13.97% higher than that of CCA-CWT-SVM, 4.74–20.04% higher than that of CCA-SVM, 5.40–21.80% higher than CCA-GNB, 8.44–26.42% higher than that of FBCCA, and 15.11–35.17% higher than CCA.

As shown in Fig. 5b, the accuracies of the different methods also increase with the increase of time window under the THU dataset. The accuracy of CBAM-CNN is up to 97.14% under the 2 s time window. Within 0.1–1 s time window, the accuracy of CBAM-CNN is 6.23–15.92% higher than that of CNN, 9.11–22.47% higher than that of CCA-CWT-SVM, 17.09–35.04% higher than that of CCA-SVM, 17.67–36.28% higher than that of CCA-GNB, 21.26–38.44% higher than that of FBCCA, and 24.04–44.00% higher than that of CCA. Within 1–2 s time window, the accuracy of CBAM-CNN is 3.32–7.02% higher than that of CNN, 4.42–9.11% higher than that of CCA-CWT-SVM, 7.66–17.09% higher than that of CCA-SVM, 8.46–17.67% higher than CCA-GNB, 9.50–21.27% higher than that of FBCCA, and 12.76–24.04% higher than CCA.

An SSVEP-BCI system usually requires high real-time performance in practical applications. For this reason, the influence of different time windows of 0.1 s, 0.5 s, 1 s, 1.5 s, and 2 s on the ITR performance for the methods is studied in this paper when the optimal $N_{L}$ is 7.

**Figure 6:**
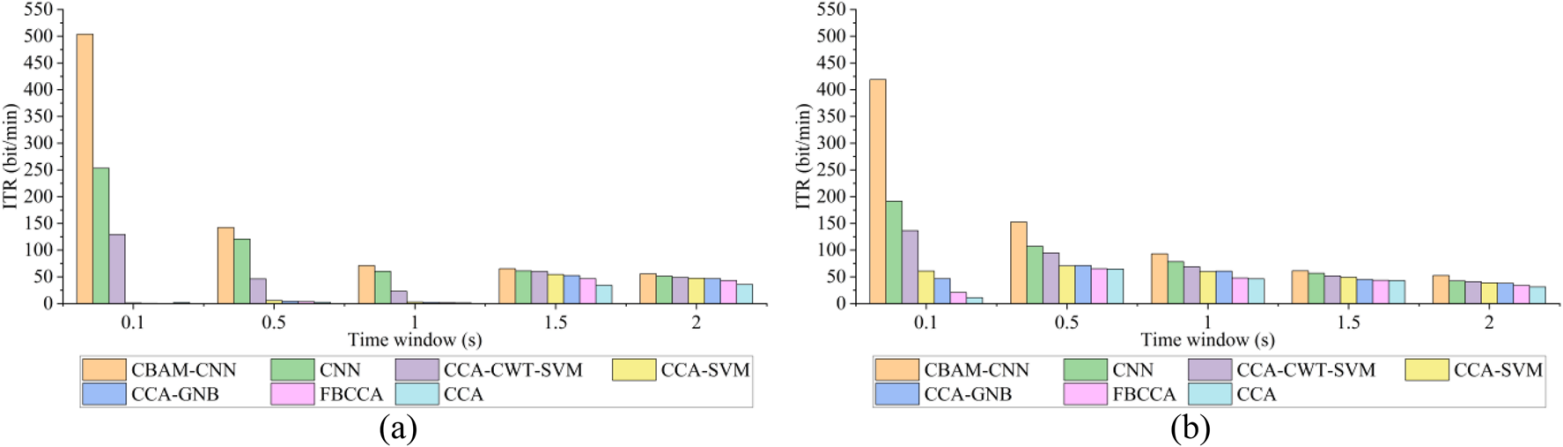
The ITRs of all methods under different time windows. **a** IMUT dataset. **b** THU dataset.

Figure 6a and b show the ITRs of different methods under IMUT and THU datasets, respectively. Under different time windows, the ITR of CBAM-CNN is always higher than the other six comparison methods. When the time window is 0.1 s, the highest ITRs of CBAM-CNN in these two datasets are 503.87 bit/min and 418.96 bit/min respectively, which are significantly higher than the other six methods. Its superior time performance shows that CBAM-CNN has a great potential to be used in various online BCI system designs.

As shown in Tables 1 and 2, the standard deviations of all classification methods in different time windows is small. There is little difference between most subjects' data and their average values in SSVEP classification method.

**Table 1:** The accuracies and standard deviations of all methods under different time windows (IMUT dataset).

Classification method	Time window				
	0.1 s	0.5 s	1 s	1.5 s	2 s
CBAM-CNN	0.736 ± 0.152	0.844 ± 0.098	0.912 ± 0.081	0.943 ± 0.060	0.981 ± 0.025
SENet-CNN	0.673 ± 0.139	0.836 ± 0.112	0.902 ± 0.048	0.920 ± 0.055	0.962 ± 0.043
SAM-CNN	0.678 ± 0.161	0.829 ± 0.099	0.895 ± 0.106	0.903 ± 0.044	0.929 ± 0.094
CNN	0.575 ± 0.162	0.789 ± 0.138	0.876 ± 0.083	0.923 ± 0.068	0.956 ± 0.049
CCA-CWT-SVM	0.500 ± 0.054	0.590 ± 0.060	0.772 ± 0.117	0.917 ± 0.070	0.945 ± 0.041
CCA-SVM	0.263 ± 0.017	0.356 ± 0.052	0.712 ± 0.147	0.882 ± 0.114	0.934 ± 0.064
CCA-GNB	0.254 ± 0.014	0.345 ± 0.039	0.694 ± 0.157	0.873 ± 0.104	0.927 ± 0.068
FBCCA	0.257 ± 0.004	0.329 ± 0.047	0.648 ± 0.173	0.830 ± 0.135	0.897 ± 0.101
CCA	0.232 ± 0.024	0.305 ± 0.054	0.560 ± 0.153	0.741 ± 0.159	0.830 ± 0.154

**Table 2:** The accuracies and standard deviations of all methods under different time windows (THU dataset).

Classification method	Time window				
	0.1 s	0.5 s	1 s	1.5 s	2 s
CBAM-CNN	0.704 ± 0.114	0.869 ± 0.083	0.928 ± 0.057	0.932 ± 0.055	0.971 ± 0.029
SENet-CNN	0.583 ± 0.107	0.796 ± 0.078	0.898 ± 0.049	0.925 ± 0.037	0.952 ± 0.052
SAM-CNN	0.591 ± 0.101	0.795 ± 0.075	0.899 ± 0.045	0.917 ± 0.038	0.930 ± 0.034
CNN	0.545 ± 0.109	0.756 ± 0.146	0.866 ± 0.130	0.899 ± 0.120	0.901 ± 0.104
CCA-CWT-SVM	0.479 ± 0.132	0.719 ± 0.165	0.837 ± 0.095	0.888 ± 0.074	0.895 ± 0.057
CCA-SVM	0.353 ± 0.143	0.618 ± 0.215	0.757 ± 0.220	0.855 ± 0.151	0.876 ± 0.119
CCA-GNB	0.341 ± 0.123	0.616 ± 0.223	0.751 ± 0.240	0.847 ± 0.160	0.871 ± 0.122
FBCCA	0.319 ± 0.071	0.615 ± 0.127	0.715 ± 0.182	0.837 ± 0.166	0.867 ± 0.138
CCA	0.264 ± 0.069	0.590 ± 0.069	0.688 ± 0.213	0.802 ± 0.204	0.844 ± 0.157

**Figure 7:**
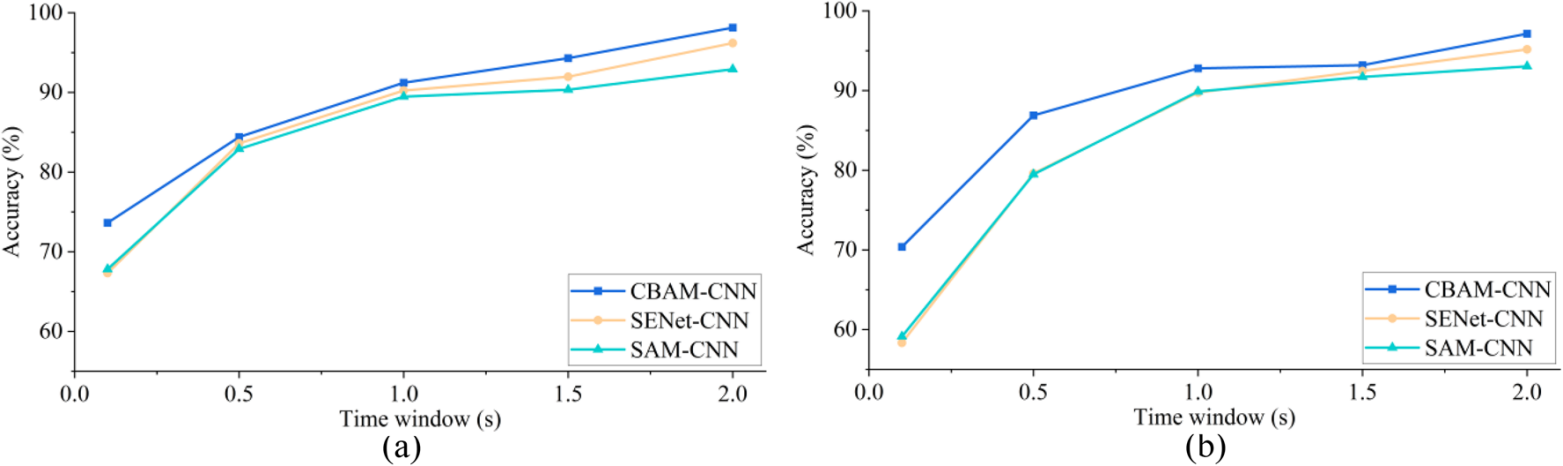
The accuracies comparison of the methods with different attention mechanisms. **a** IMUT dataset. **b** THU dataset.

To verify the effectiveness and advantages of CBAM used in CBAM-CNN, this paper compares the performance of the methods when CBAM, Spatial Attention Mechanism (SAM) and SENet attention mechanism are selected. Among them, SAM belongs to spatial attention mechanism and SENet belongs to channel attention mechanism. All experiments were carried out on the premise of $N_{L} = 7$. The SENet-CNN and CAM-CNN are obtained by replacing only the attention mechanism in CBAM-CNN. As shown in Fig. 7, the accuracy of CBAM-NN in different time windows is 0.73–12.05% and 1.5–11.27% higher than that of SENet-CNN and SAM-CNN, respectively.

Additionally, CNN and CBAM-CNN are also compared in comprehensive performance ($N_{L} = 7$). Table 3 shows the performance of different methods when using IMUT dataset. Under different time windows, the accuracy of CBAM-CNN is 2.01–16.17% higher than that of CNN, the precision of CBAM-CNN is 0.81–13.14% higher than that of CNN, the recall of CBAM-CNN is 0.39–14.45% higher than that of CNN, and the macro-F1 of CBAM-CNN is 0.39–15.63% higher than that of CNN.

**Table 3:** Comparison of comprehensive performance metrics between CNN and CBAM-CNN.

SSVEP dataset	Performance metric	Classification method	Time window				
			0.1 s	0.5 s	1 s	1.5 s	2 s
THU dataset	Macro-F1	CBAM-CNN	0.68397	0.85404	0.91675	0.92242	0.96652
		CNN	0.54047	0.75359	0.86340	0.89474	0.89891
	Precision	CBAM-CNN	0.70193	0.86064	0.92720	0.93619	0.96973
		CNN	0.55241	0.76603	0.87911	0.90839	0.92462
	Recall	CBAM-CNN	0.68645	0.85498	0.91849	0.92423	0.96707
		CNN	0.54432	0.75608	0.86586	0.89822	0.90110
	Accuracy	CBAM-CNN	0.70387	0.86887	0.92793	0.93185	0.97139
		CNN	0.54463	0.75594	0.86563	0.89868	0.90120
IMUT dataset	Macro-F1	CBAM-CNN	0.71734	0.83048	0.90358	0.93717	0.97721
		CNN	0.56109	0.78428	0.89403	0.93326	0.95630
	Precision	CBAM-CNN	0.72177	0.85868	0.91551	0.94558	0.97775
		CNN	0.59039	0.80114	0.90287	0.93744	0.95881
	Recall	CBAM-CNN	0.71916	0.83308	0.90460	0.93693	0.97710
		CNN	0.57469	0.78806	0.89589	0.93308	0.95609
	Accuracy	CBAM-CNN	0.73624	0.84401	0.91208	0.94294	0.98131
		CNN	0.57454	0.78872	0.87580	0.92280	0.95626

It also can be seen from Table 3 that the performance of different methods when using THU dataset. Under different time windows, the accuracy of CBAM-CNN is 3.32–15.92% higher than that of CNN, the precision of CBAM-CNN is 2.78–14.95% higher than that of CNN, the recall of CBAM-CNN is 2.60–14.21% higher than that of CNN, and the macro-F1 of CBAM-CNN is 2.77–14.35% higher than that of CNN.

According to the comprehensive evaluation of accuracy, precision, recall, and macro-F1 in Table 3, it can be seen that the classification performance of CBAM-CNN is obviously better than that of CNN.

The *P*-values of CBAM-CNN and other methods are presented in Table 4 of the manuscript. The *P*-values in Table 4 are all less than 0.05, which confirms the significance of CBAM-CNN compared to other methods.

**Table 4:** *P*-value of CBAM-CNN and different comparison methods for statistical tests.

SSVEP dataset	Classification method	Time window				
		0.1 s	0.5 s	1 s	1.5 s	2 s
IMUT dataset	SENet-CNN	0.000779	0.003685	0.001174	0.005816	0.011005
	CNN	0.000040	0.002023	0.007232	0.008069	0.001341
THU dataset	SENet-CNN	0.000072	0.000811	0.002399	0.004350	0.043050
	CNN	0.000012	0.009842	0.001107	0.001098	0.000811

## Conclusion

In the SSVEP-BCI paradigm, the proposed CBAM-CNN method can identify the target frequencies of SSVEP signals with high performance metrics such as accuracy, ITR, recall, precision, and macro-F1. The CBAM-CNN method firstly extracts four subfrequency band signals by a Butterworth filter. The multi-subfrequency bands signals are processed by three convolution layers and CBAM module to obtain four refinement features. Then, the feature fusion layer performs feature fusion on the four refinement features. In addition, the CBAM-CNN method also embeds a second CBAM module between the feature fusion layer and the Conv4 layer. The second CBAM module is used to enhance the concentration of important features in space and channel latitude. Four subfrequency band signals can provide more useful information and filter disturbance information for the brain networks. Thus, the multi-subfrequency bands and CBAM can make CBAM-CNN better realize the target frequency classification of SSVEP signals. The experiment uses IMUT and THU datasets to train and test the classification tasks. The seven leads in the occipital area and parietal lobes area of the brain including O1, O2, P3, P4, PZ, P7, and P8 are used to capture the SSVEP signals. The experimental results show that the accuracies of the seven EEG decoding methods, CBAM-CNN, CNN, CCA-CWT-SVM, CCA-SVM, CCA-GNB, FBCCA, and CCA, all increase with the increase of $N_{L}$. Within the time window of 0.1–2 s, the accuracy of CBAM-CNN reaches 98.13%, which is 2.01–16.17%, 2.54–25.38%, 4.74–48.85%, 5.40–49.94%, 8.44–47.89% and 12.76–53.88% higher than that of CNN, CCA-CWT-SVM, CCA-SVM, CCA-GNB, FBCCA, and CCA. The highest ITR of CBAM-CNN is 503.87 bit/min, which is 227.53 bits/min-503.41 bit/min higher than the other six methods. Especially, the performance of CBAM-CNN is more significant when the short time window is 0.1–1 s. When the time window is 0.1 s, the accuracy of CBAM-CNN is 73.62% and ITR is 503.87 bit/min. The significant short-time performance provides the possibility for the application of embedded real-time BCI systems, such as brain-controlled wheelchairs. In addition, compared with the classical CNN, CBAM-CNN has significantly higher performance metrics in macro-F1, precision, recall, and accuracy, which are 0.39–15.63%, 0.81–14.95%, 0.39–14.45%, and 2.01–16.17% respectively. The above performance metrics show the effectiveness of the proposed CBAM-CNN method.

## Data Availability

The data that support the findings of this study are included in the article. Further information is available from the corresponding author [C.D.] upon reasonable request.
